# Hispidin in the Medicinal Fungus Protects Dopaminergic Neurons from JNK Activation-Regulated Mitochondrial-Dependent Apoptosis in an MPP^+^-Induced In Vitro Model of Parkinson’s Disease

**DOI:** 10.3390/nu15030549

**Published:** 2023-01-20

**Authors:** Mei-Chou Lai, Wayne-Young Liu, Shorong-Shii Liou, I-Min Liu

**Affiliations:** 1 Department of Pharmacy and Master Program, Collage of Pharmacy and Health Care, Tajen University, Pingtung County 90741, Taiwan; 2 Department of Urology, Jen-Ai Hospital, Taichung 41265, Taiwan; 3 Center for Basic Medical Science, Collage of Health Science, Central Taiwan University of Science and Technology, Taichung City 406053, Taiwan

**Keywords:** Parkinson’s disease, hispidin, medicinal fungus, 1-methyl-4-phenylpyridinium, MS23.5 cells

## Abstract

Degenerative diseases of the brain include Parkinson’s disease (PD), which is associated with moveable signs and is still incurable. Hispidin belongs to polyphenol and originates primarily from the medicinal fungi Inonotus and Phellinus, with distinct biological effects. In the study, MES23.5 cells were induced by 1-methyl-4-phenylpyridinium (MPP^+^) to build a cell model of PD in order to detect the protective effect of hispdin and to specify the underlying mechanism. Pretreatment of MES23.5 cells with 1 h of hispdin at appropriate concentrations, followed by incubation of 24 h with 2 μmol/L MPP^+^ to induce cell damage. MPP^+^ resulted in reactive oxygen species production that diminished cell viability and dopamine content. Mitochondrial dysfunction in MS23.5 cells exposed to MPP^+^ was observed, indicated by inhibition of activity in the mitochondrial respiratory chain complex I, the collapse of potential in mitochondrial transmembrane, and the liberation of mitochondrial cytochrome c. Enabling C-Jun N-terminal kinase (JNK), reducing Bcl-2/Bax, and enhancing caspase-9/caspase-3/PARP cleavage were also seen by MPP^+^ induction associated with increased DNA fragmentation. All of the events mentioned above associated with MPP^+^-mediated mitochondrial-dependent caspases cascades were attenuated under cells pretreatment with hispidin (20 µmol/L); similar results were obtained during cell pretreatment with pan-JNK inhibitor JNK-IN-8 (1 µmol/L) or JNK3 inhibitor SR3576 (25 µmol/L). The findings show that hispidin has neuroprotection against MPP^+^-induced mitochondrial dysfunction and cellular apoptosis and suggest that hispidin can be seen as an assist in preventing PD.

## 1. Introduction

Parkinson’s disease (PD) is the second most frequent neurodegenerative disorder that predominantly influences dopamine (DA) production areas of substantia nigra (SA) [1]. Most cases of PD occur sporadically, but approximately 10–15% of patients have family histories of PD [1]. The main features of PD are four motor symptoms, including resting tremors, bradykinesia, stiffness, and postural instability [1]. While current therapies are intended to relieve symptoms and slow the progression of PD, the harmful motor complications of current PD drugs are still possible [2]. Several ongoing studies are aimed at finding effective treatments with fewer side effects.

Numerous factors participate in the pathologic process of degeneration of DA neurons; most of this is oxidative stress, resulting from a disruption of the equilibrium between reactive oxygen species (ROS) generation and the antioxidant defense of the cell [3]. The human antioxidant defense system is mainly composed primarily of superoxide dismutase (SOD), glutathione peroxidase (GSH-Px), and catalase (CAT) [4]. Reduced activity of several antioxidant enzymes was observed in SA of the Parkinsonian brain [3]. Mitochondria are known to produce approximately 90% ROS within cells [5]. Accumulation of ROS may activate several stress-sensitive serine kinase cascades, including c-Jun-N-terminal kinase (JNK), which is one of the mitogen-activated protein kinase (MAPK) [6]. The mitochondrial transmembrane potential (ΔΨM) is subsequently destroyed upon activation of JNK, causing cytochrome c release to activate the downstream caspases, which eventually leads to apoptosis involving the progression of PD [7]. It may be reasonably inferred to lessen the complex factors behind neurodegeneration, such as mitochondrial ROS/JNK/caspase signaling dysfunction, as discussed above, could be a therapeutic strategy for PD [8].

Hispidin, 6-(3,4-dihydroxystyryl)-4-hydroxy-2-pyrone, is one of the phenolic substances widely distributed in edible and medicinal mushrooms of the Phellinus and Inonotus genera [9]. *Inonotus* and *Phellinus* are two genera in the Hymenochaetaceae that have been commonly used to treat a variety of disorders, such as cancers, heart, and hepatic disorders, diabetes, etc., in traditional medicine [10]. Hispidin is a promising bioactive compound due to its antioxidant potency and possesses multiple biological effects, including anti-inflammatory and antitumor actions [11,12,13]. Hispidin has anti-diabetic properties because of its powerful inhibitory activity of protein glycation [14]. In fact, hispidin has significant neuroprotective properties. Hispidin has been documented to inhibit the generation of hydroxyl radicals against peroxynitrite-induced DNA lesions in primary rat astrocytes, the most abundant cells in the central nervous system [15]. Hispidin inhibits ß-site amyloid precursor protein cleaving enzyme 1 to reduce the accumulation of ß-amyloid peptide [16]. In addition, hispidin has been shown to inhibit nitric oxide generation caused by lipopolysaccharide in BV-2 cells that depend on MAPK signaling linked to ROS. [17]. Although the above results provide additional guidance on the possibility that hispidin may serve as a targeted treatment to improve neuroinflammation or neural degeneration caused by oxidative stress, the protection of hispidin from PD is not fully apparent, and possible mechanisms need to be strongly clarified.

Cellular models generally develop pathology faster, more cost-effectively, and do not require ethical approval in comparison with animal models [18]. Cellular models are therefore ideal for large-scale drug detection that may help focus on potential drug targets for later validation in animal models [18]. A variety of neurotoxicity and genetic experimental models have been developed for PD studies [18]. Neurotoxin-based models led to the rapid degeneration of nigrostriatal dopaminergic neurons, which mimic sporadic PD [19]. The 1-methyl-4-phenyl-1,2,3,6-tetrahydropyridine (MPTP) neurotoxin is known to be transformed into an active metabolite 1-methyl-4-phenylpyridinium (MPP^+^) to damage the catecholaminergic neurons of the brain, resulting in PD-like motor symptoms [20]. As a result, MPTP is currently the most useful and practical model for PD studies [20]. MES23.5 cells have a number of characteristics similar to those of SN-derived primary neurons [21]. Therefore, MPP^+^ was used to induce a PD-like model in MES23.5 cells to provide an obvious direct correlation of hispidin on degenerated dopamine neurons with the mechanism of action.

## 2. Materials and Methods

### 2.1. Cell Culture and Treatment

MS23.5 cells acquired from the American Type Culture Collection (Manassas, VA, USA) were grown in DMEM/F12 (Sigma-Aldrich, St. Louis, MO, USA) with Sato’s components with 10% (*v*/*v*) fetal bovine serum, including 100 U/mL penicillin and 100 mg/mL streptomycin. Cells were grown at 37 °C in a humid environment with 5% CO_2_ and 95% air. In the experiments, the cells were seeded into plates of 6 wells at a density of 1 × 10^5^ cells per well, and cultures were separated into 0.05% (*w*/*v*) trypsin in a saline phosphate buffer (PBS) at a pH of 7.4 at the confluence.

For the establishment of the PD model, MES23.5 cells were treated with MPP^+^ (Sigma-Aldrich, St. Louis, MO, USA; Cat. # M0896) at a concentration of 0.5–2.5 mmol/L at 37 °C over a 24-h period. In the pretreatment studies, cells were incubated for 1 h with either hispidin at the indicated concentrations (5, 10, 20, and 40 µmol/L) or 1 µmol/L JNK-IN-8 (Sigma Chemical Co., St. Louis, MO, USA; Cat. # SML1246) and 25 µmol/L SR3576 (Sigma Chemical Co., St. Louis, MO, USA; Cat. # S8201), after exposure to 2 mmol/L of MPP^+^ for 24 h. Untreated MES23.5 cells cultured during 24 h at 37 °C in normal conditions were used as controls. The stock solution of the experimental compounds at 1 mmol/L was prepared by dimethyl sulfoxide (DMSO, Sigma-Aldrich, St. Louis, MO, USA; Cat. # D8418) as a solvent, which was diluted in a culture medium at concentrations adequate for the following study. The final DMSO concentration was less than 0.1% (*v*/*v*), resulting in no toxicity to most cells [22].

### 2.2. Cell Viability Assay

Cell viability was determined based on the Cell Counting Kit-8 (CCK-8) test (Cat. 96992) following the manufacturer’s protocol (Sigma Chemical Co., St. Louis, MO, USA). The cells were inoculated in 96-well plates with a density of 2 × 10^4^ cells/mL. After treatment, 10 μL of the CCK-8 reagent was added to each well, and the absorbance value of each well at 450 nm was measured with a multifunction microplate reader (SpectraMax M5, Molecular Devices, Sunnyvale, CA, USA) after incubation for 2 h at 37 °C. Cells treated with the vehicle were considered controls, and cell viability was defined to be 100%. The viability of the treatment group was expressed in percent of the control group.

### 2.3. Determination of DA

The DA levels were detected through high-performance liquid chromatography (HPLC) using a fluorescence detector (Waters 2475, Milford, MA, USA). Briefly, cells were taken after treatment and then were sonicated in 0.2 mol/L perchloric acid with isoproterenol, and homogenates obtained were centrifuged at 20,000× *g* for 15 min under 4 °C. The supernatant was then collected and filtered through a 0.22 μm filter, and 25 μL of the sample was injected into the column. An Atlantis T3 column (150 mm × 4.6 mm, 5 μm, Waters) was employed in the separation system (Waters 2695). The mobile phase has a flow rate of 0.6 mL/min, composed of acetonitrile and water (90:10% *v*/*v*) and 75 mmol/L pH 3.0 phosphate buffer containing octane sulfonic acid 1.8 mmol/L, EDTA 30 µmol/L, and triethylamine 0.015% (*v*/*v*). The standard solution was prepared fresh by dilution of the mobile phase stock solution. Serial concentrations of the standard solution determined the linearity ranges of DA before detection. Results were reported in ng/10^6^ cells.

### 2.4. Cellular ROS Assay

The ROS level of the cell was measured with dichloro-dihydro-fluorescein diacetate (DCFH-DA), as instructed by the manufacturer (Sigma-Aldrich, St. Louis, MO, USA) [23]. In summary, DCFH-DA was diluted with DMEM at a final concentration of 10 μmol/L and then added to the cells for 20 min incubating at 37 °C. The cells were washed 3 times with PBS after treatment with DCFH-DA. A phase contrast fluorescence microscope (Nikon, model 80i) was used to capture the images. The DCF fluorescence was read from a multifunctional microplate reader (SpectraMax M5, Molecular Devices, Sunnyvale, CA, USA) with 488 nm excitation and 525 nm emission wavelengths. The increase in ROS levels was expressed as a percentage of control.

### 2.5. Antioxidant Enzyme Activity Assay

The following commercial kits, based on an enzyme-linked immunosorbent assay (ELISA), were used to estimate antioxidant activity. The Activity Colorimetric Assay Kits of SOD (Cat. # K335), GSH-Px (Cat. # K762), and CAT (Cat. # K773) were purchased from Bio Vision, Inc. (San Francisco, CA, USA). The activities of SOD, GSH-Px, and CAT were calculated to measure the absorbance recorded at 450 nm, 340 nm, and 570 nm, respectively, by a microplate reader (SpectraMax M5, Molecular Devices, Sunnyvale, CA, USA). Enzymatic activities were normalized from the respective protein concentration of each group, expressed in units per milligram of protein. The protein level was quantified using a Bradford protein assay.

### 2.6. Mitochondrial Transmembrane Potential (ΔΨM) Assay

The ΔΨM was measured using an assay kit containing 5,5′,6,6′-tetrachloro-1,1′,3,3′-tetraethylbenzimi-dazolylcarbocyanin iodine (JC-1) dye (Abcam plc., Cambridge, MA, USA). Cells were seeded into a 96-well plate at a density of 1 × 10^4^ cells/well, which was incubated with 20 µmol/L JC-1 at 37 °C for 30 min. The cells were then centrifuged at 2500 rpm for 5 min, and the pellets were resuspended in 0.5 mL PBS. The red aggregates emit at 590 nm, and the green-fluorescent monomer with a 530 nm emission was determined by a fluorescence spectrophotometer (SpectraMax M5, Molecular Devices, Sunnyvale, CA, USA). The ratio of J-aggregates to J-monomers was computed to determine the changes in the ΔΨM [24]. The ratio of fluorescence intensity of J aggregates to the fluorescence intensity of monomers was used as an indicator to determine changes in ΔΨM.

### 2.7. Measurement of ADP and ATP Levels

The commercially available assay kit (Cat. # ELDT-100) from BioAssay Systems (Hayward, CA, USA) was used to determine the ability of luciferase to produce light in the presence of its luciferal substrate [25]. After treatment, cells were lysed with 10% tricholoroacetic acid, then neutralized with 1 mol/L of KOH, and diluted with 100 mmol/L HEPES buffer (pH 7.4). The first step in the test was the luciferase-catalyzed response of cellular ATP and D-luciferin to produce a luminescent signal. Later, ADP was converted into ATP by enzyme reaction, while the newly formed ATP responded with D-luciferin. The second light intensity accounted for the total amount of ADP and ATP. The ratio of ADP/ATP was normalized based on the total protein content of the samples.

### 2.8. Measurement of Cytochrome C Release

The cells were homogenized following treatment. The lysate spun two times at 800× *g* for 20 min. The mitochondrial granule was produced from the supernatant after centrifugation at 10,000× *g* over a period of 15 min. The remaining supernatant was centrifuged at 16,000× *g* within 25 min for a cytosolic fraction. The cytochrome C ELISA kit was used to measure the cytochrome C level in mitochondria and cytosolic fraction as indicated by the manufacturer. Cytochrome c is immune-captured inside the wells, determined by the addition of a specific cytochrome c antibody conjugated to horseradish peroxidase. This peroxidase changes the substrate from colorless to blue, which was measured at 450 nm. The protein content was measured with Bio-Rad protein analysis.

### 2.9. Analyses of Mitochondrial Complex I Activity

The commercial ELISA kit (Abcam plc., Cambridge, MA, USA; Cat. AB109721) was used to detect the activity of mitochondrial complex 1. The protein concentration was adjusted to 1 mg/mL in an incubation solution following cell lysis. The 200 μL diluted sample and the control were charged into the wells with a microplate pre-coated with complex I capture antibodies for 3 h at room temperature. Complex I activity was determined by measuring the oxidation of NADH to NAD+ and the simultaneous reduction of a dye, resulting in enhanced absorption at 450 nm. The activity of complex I was expressed as nmol oxidized NADH/min/mg protein. Protein contents were measured with a Bio-Rad protein assay.

### 2.10. Analysis of the Levels of Activated JNK and c-Jun, and the Expressions of Bax and Bcl-2

The phosphorylation status of JNK and c-Jun was determined using the Phospho-JNK1/2/3 (Thr183 + Tyr185) Colorimetric Cell-Based ELISA Kit (Cat. # CBCAB00421) and c-Jun (Phospho-Ser63) Colorimetric Cell-Based ELISA kit (Cat. # CBCAB00581), respectively. The protein amounts of Bcl-2 (Cat. # CBCAB00158) and Bax (Cat. # CBCAB00157) were also measured with commercial ELISA kits. The listed ELISA kits were purchased from Assay Genie (Windsor Place, Dublin, Ireland) and performed as per the manufacturer’s protocols. The commercially available kit is based on primary target-specific antibodies and was detected by a secondary antibody conjugated with horseradish peroxidase. The optical density (OD) was determined on the target absorbance values at a wavelength of 450 nm with the aid of a microplate reader (SpectraMax M5, Molecular Devices, Sunnyvale, CA, USA).

### 2.11. Measurement for Activities of Caspases and Poly (ADP-Ribose) Polymerase (PARP)

The activities of caspase-9 and caspase-3 were determined using the caspase colorimetric test kits based on spectrophotometric detection of the chromophore p-nitroanilide (p-NA) as a result of the cleavage of the labeled substrate acetyl-Leu-Glu-His-Asp-pNA and Asp-Glu-Val-Asp-pNA, respectively. The assay kits for caspases-9 (Cat. # ab65608) and caspase-3 (Cat. # ab39401) were purchased from Abcam plc. (Cambridge, MA, USA). Free pNA was identified with a microplate reader (SpectraMax M5, Molecular Devices, Sunnyvale, CA, USA) at 405 nm.

The PARP/Apoptosis colorimetric assay kit (R&D Systems, Minneapolis, MN, USA; Cat. # 4684-096-K) was used for the determination of PARP activity based on a semi-quantitative measurement of the amount of poly (ADP-ribose) deposited on the immobilized histone proteins. The absorbance values at 450 nm were obtained from a microplate reader (SpectraMax M5, Molecular Devices, Sunnyvale, CA, USA). All values were compared to the controls treated by the vehicle.

### 2.12. Study of Apoptotic DNA Fragmentation

DNA fragments associated with cytoplasmic histones resulting from induced cell death were quantitatively detected through the cell death detection ELISA kit (Roche Molecular Biochemicals, Mannheim, Germany; Cat. #11774425001). Cytoplasmic cell extracts were treated with a primary mouse anti-histone monoclonal antibody coated with a microtiter plate and then a second mouse anti-DNA monoclonal antibody coupled with peroxidase. The quantity of peroxidase retained in the immune complex was determined by photometric analysis by incubation with 2,2′-azino-di-[3-ethylbenzthiazoline sulfonate] as a substrate for 10 min at 20 °C. The color shift was measured at 405 nm with a microplate reader (SpectraMax M5, Molecular Devices, Sunnyvale, CA, USA).

### 2.13. Statistical Analysis

Data are expressed as the mean ± standard deviation (SD). Statistical analysis was performed using Systat SigmaPlot version 14.0. (Systat Software Inc., San Jose, CA, USA). Three wells were tested in each experiment, and each experiment was performed a minimum of five times. Where no special description is available, significant differences from the vehicle controls were evaluated using one-way ANOVA analysis followed by Dunnett’s test as a post-hoc test. The differences were statistically significant at *p* < 0.05.

## 3. Results

### 3.1. Hispidin Alleviates MPP^+^-Induced Loss of Cell Viability and DA Content

**Figure 1:**
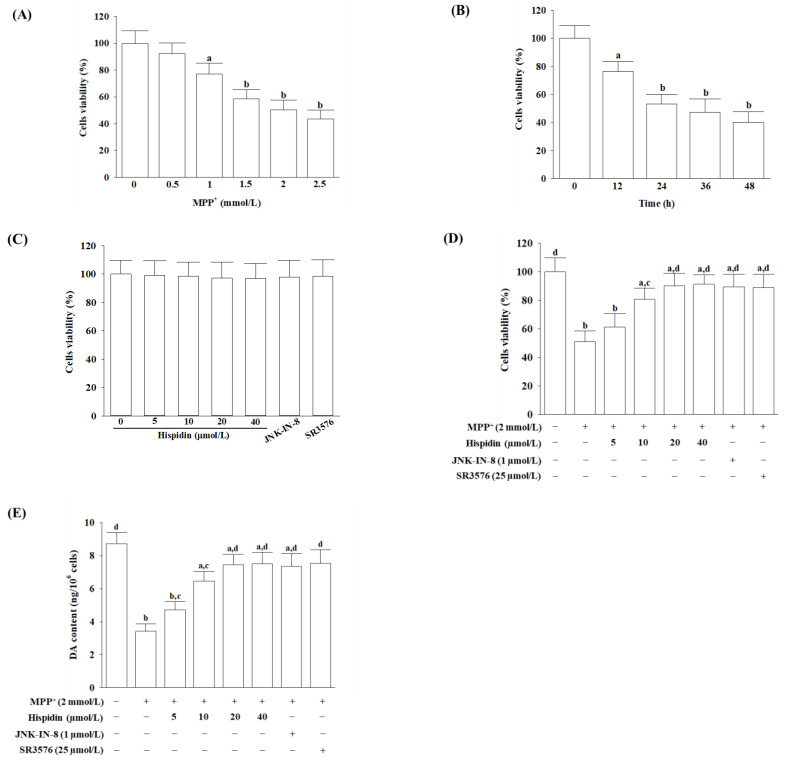
Hispidin alleviates MPP^+^-induced loss of cell viability and DA content in MES23.5 cells. (**A**) Cell viability was assessed for MES23.5 cells incubated with increasing concentrations of MPP^+^ over 24 h. (**B**) Cell viability was evaluated for MES23.5 cells incubated with 2 mmol/L MPP^+^ over a given period. (**C**) Cell viability was evaluated when MES23.5 cells without stimulation of 2 mmol/L MPP^+^ instead of 24-h incubation at various concentrations of hispidin, JNK-IN-8 (1 µmol/L), or SR3576 (25 µmol/L). (**D**) Cell viability and (**E**) dopamine (DA) content was evaluated when MES23.5 cells received 1 h pretreatment with hispidin at different concentrations, JNK-IN-8 (1 µmol/L) or SR3576 (25 µmol/L), and following received 2 mmol/L MPP^+^ exposure for another 24 h. Cell viability was determined with a CCK-8 assay and expressed as a percentage of untreated cells taken as a control group. DA content as determined by HPLC assay. The results are shown as the mean ± SD of five independent experiments (*n* = 5) performed in triplicate. ^a^ *p* < 0.05 and ^b^ *p* < 0.01 compared to the untreated control group. ^c^ *p* < 0.05 and ^d^ *p* < 0.01 compared to the untreated MPP^+^ group.

In order to realize the relation between concentration and time in cytotoxicity induced by MPP^+^, cell viability was identified after cells were incubated with MPP^+^ at concentrations of 0.5–2.5 mmol/L at 12–48 h. (Figure 1A,B). A significant reduction in cell viability was observed when MES23.5 cells were incubated for 24 h with an increasing concentration of MPP^+^ (Figure 1A). A reduction in cell viability over time was observed by 2 mmol/L MPP^+^ (Figure 1B). The 50% cell inhibition was obtained by incubation of the cell with MPP^+^ at 2 mmol/L for 24 h (Figure 1B). Based upon the above findings, MES23.5 cells were exposed 24 h to 2 mmol/L MPP^+^ to cause a cellular lesion in later experiments.

The viability of MES23.5 cells was not influenced by hispidin at 5–40 µmol/L (Figure 1C). Cell death does not occur with 1 µmol/L JNK-IN-8 or 25 µmol/L SR3576 (Figure 1C). Hispidin inhibits MPP^+^-induced cell death according to concentration (Figure 1D). Cell viability in cells stimulated by MPP^+^ was 89.5% and 88.9% for 1 h pretreated with 1 µmol/L JNK-IN-8 and 25 µmol/L SR3576, respectively (Figure 1D).

A noticeable reduction in the DA content of cellular lysates was detected following a 24-h exposure with MPP^+^ (2 mmol/L), while pretreatment with hispidin increased DA levels in MPP^+^-induced cells in proportion to concentration (Figure 1E). The pretreatment cell with 1 µmol/L JNK-IN-8 or 25 µmol/L SR3576 eliminated the MPP^+^-induced AD reduction (Figure 1E).

### 3.2. Hispidin Alleviated MPP^+^-Induced Oxidative Stress

**Figure 2:**
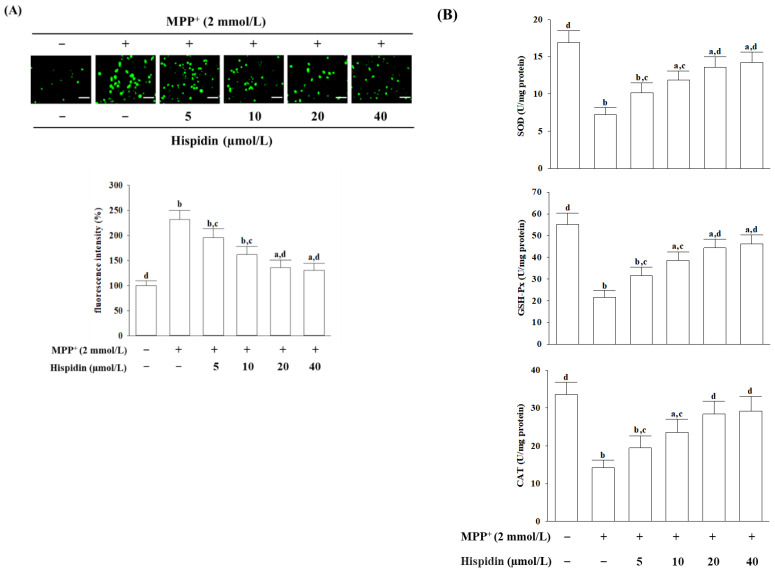
Hispidin protects MES23.5 cells against MPP^+^-activated oxidative stress. MES23.5 cells were pretreated with hispidin at the indicated concentration for 1 h and then exposed to 2 mmol/L MPP^+^ for another 24 h. (**A**) The image-based measure of ROS in MES23.5 cells was captured by fluorescence microscopy. Scale bar, 50 μm. The ROS fluorescence intensity is expressed as a percentage of the untreated control cells. (**B**) SOD, GSH-Px, and CAT activities were normalized to the corresponding protein concentration for each group and expressed in units per milligram of protein. The results are shown as the mean ± SD of five independent experiments (*n* = 5) performed in triplicate. ^a^ *p* < 0.05 and ^b^ *p* < 0.01 compared to the untreated control group. ^c^ *p* < 0.05 and ^d^ *p* < 0.01 compared to the untreated MPP^+^ group.

An image reflected on the ROS in MES23.5 cells was captured by fluorescence microscopy (Figure 2A). ROS fluorescence intensity shows that MPP^+^ elevated intracellular ROS levels in MES23.5 cells to 2.4-fold of untreated controls (Figure 2A). Hispidin lowered the MPP^+^-induced intracellular ROS level with concentrations (Figure 2A). A 40.4% reduction in MPP^+^-induced ROS production was observed when MES23.5 cells were pretreated with 20 µmol/L hispidin (Figure 2A).

The activities of SOD, GSH-Px, and CAT were drastically reduced in MES23.5 cells receiving MPP^+^ induction; it has been improved by hispidin under dependent concentration (Figure 2B). MPP^+^ reduced intracellular activities in SOD, GSH-Px, and CAT, which were increased to 1.8-, 2.2-, and 2.0-fold, respectively, when MES23.5 cells were given 20 µmol/L hispidin pretreatment (Figure 2B).

### 3.3. Hispidin Prevents Mitochondrial Dysfunction from MPP^+^

**Figure 3:**
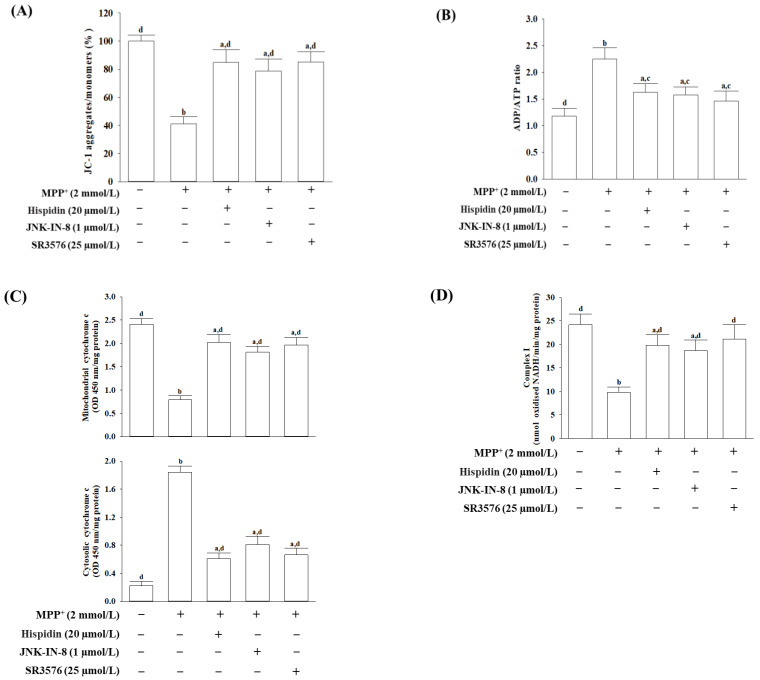
Hispidin prevents MS23.5 cells from MPP^+^-mediated mitochondrial dysfunction. MS23.5 cells were pretreated with hispidin (20 µmol/L), JNK-IN-8 (1 µmol/L), or SR3576 (25 µmol/L) for 1 h and then exposed to 2 mmol/L MPP^+^ for another 24 h. (**A**) ΔΨM has been measured with the JC-1 fluorescence probe. (**B**) The ADP/ATP ratio in cells was measured using a commercial assay kit based on the bioluminescent detection of ADP and ATP levels. (**C**) Cytochrome c concentrations were determined by immunoassay for mitochondrial and cytosolic fractions. (**D**) The activity of the mitochondrial complex I was determined by measuring the oxidation of NADH. The results are shown as the mean ± SD of five independent experiments (*n* = 5) performed in triplicate. ^a^ *p* < 0.05 and ^b^ *p* < 0.01 compared to the untreated control group. ^c^ *p* < 0.05 and ^d^ *p* < 0.01 compared to the untreated MPP^+^ group.

ΔΨM in MES23.5 cells treated with MPP^+^ was decreased to 41.2% of controls in untreated vehicles (Figure 3A). Hispidin (20 µmol/L) attenuated MPP^+^-induced ΔΨM disturbance in MES23.5 cells (Figure 3A). Reduction of ΔΨM in MES23.5 cells following MPP^+^ was also elevated in the pretreated JNK-IN-8 (1 μmol/L) or SR3576 (25 µmol/L) group (Figure 3A).

The 2.1-fold increase in ADP/ATP ratio was achieved in MPP^+^-stimulated MES23.5 cells compared with controls, which was reduced by pretreatment of hispidin (20 μmol/L) with a 27.5% reduction (Figure 3B). The ADP/ATP ratio in MES23.5 cells pretreated JNK-IN-8 (1 μmol/L) or SR3576 (25 µmol/L) was less than 29.7% and 35.1%, respectively, of the MPP^+^ induction values (Figure 3B).

Cytochrome c levels in the mitochondrial fraction decreased, but in the cytosolic fraction was a parallel increase in MPP^+^-inducing MES23.5 cells (Figure 3C). The MPP^+^-induced cytochrome c liberation of mitochondria in the cytoplasm was attenuated when MES23.5 cells received hispidin (20 μmol/L) pretreatment (Figure 3C). If pretreatment of MES23.5 cells with JNK-IN-8 (1 μmol/L) or SR3576 (25 µmol/L) follows MPP^+^ induction, the release of cytochrome c from mitochondria to the cytoplasm is reduced (Figure 3C).

MPP^+^ made the activity of mitochondrial chain I complexes in MES23.5 cells to be 42.2% lower than the control (Figure 3D). If the cell is treated with hispidin (20 μmol/L) after MPP+ induction, the activity of the mitochondrial complexes I has been increased to 82.1% of the untreated control (Figure 3D). The MPP^+^ reduction in the mitochondrial chain I complex activity was mitigated when the cell was pretreated with 1 µmol/L JNK-IN-8 (74.6% of the untreated control) or 25 μmol/L SR3576 (87.4% of the untreated control; Figure 3D).

### 3.4. Hispidin Reduces MPP^+^-Induced JNK/Bcl-2 Pathway

**Figure 4:**
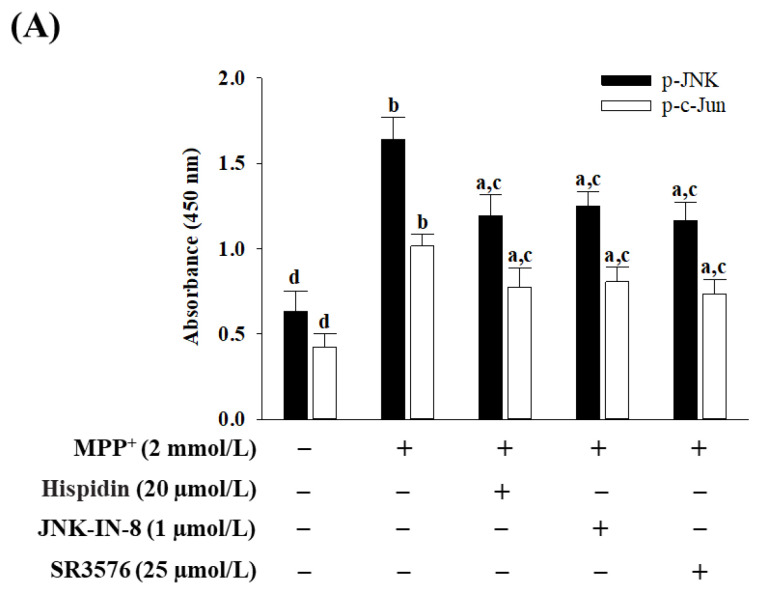

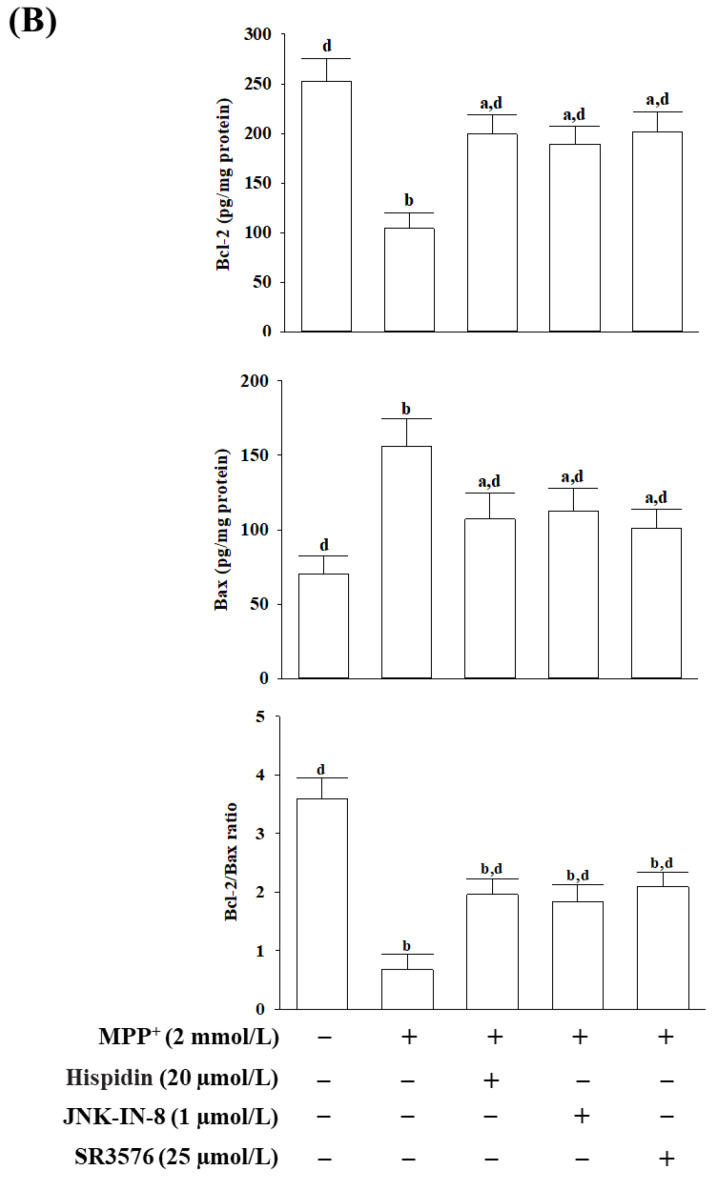
Hispidin reduces JNK-associated Bcl-2 pathway mediated by MPP^+^. MS23.5 cells were pretreated with hispidin (20 µmol/L), JNK-IN-8 (1 µmol/L), or SR3576 (25 µmol/L) for 1 h and then exposed to 2 mmol/L MPP^+^ for another 24 h. (**A**) Phosphorylation of JNK and c-Jun has been detected in ELISA kits available on the market. (**B**) Levels of Bcl-2 and Bax were measured through ELISA with commercially available kits. The ratio of relative intensities in Bcl-2 to Bax (Bcl-2/Bax) was reported. The results are shown as the mean ± SD of five independent experiments (*n* = 5) performed in triplicate. ^a^ *p* < 0.05 and ^b^ *p* < 0.01 compared to the untreated control group. ^c^ *p* < 0.05 and ^d^ *p* < 0.01 compared to the untreated MPP^+^ group.

Phosphorylation of JNK and c-Jun in cells receiving MPP^+^ induction was 2.6- and 2.4-fold higher than in controls, respectively (Figure 4A). The hispidin (20 μmol/L) pretreatment cells reduced MPP^+^-induced higher phosphorylation of JNK and c-Jun at 27.1 and 23.6%, separately. JNK phosphorylation was reduced by 24.1%, and C-Jun phosphorylation was reduced by 20.6% in cells pretreated with JNK-IN-8 (1 μmol/L) after exposure to MPP+; similar results were obtained if the cell was pretreated with 25 μmol/L of SR3576 (29.1% reduction in JNK phosphorylation and 27.4% reduction in c-Jun phosphorylation; Figure 4A).

The MPP^+^ treatment decreased the Bcl-2 protein content and increased the Bax protein content, thereby reducing the Bcl-2/Bax ratio (Figure 4B). Pretreatment with hispidin (20 μmol/L) reversed the MPP^+^ induction on the Bcl-2 protein reduction and the Bax protein increase, thus showing the high Bcl-2/Bax ratio (Figure 4B). Pretreatment cells with JNK-IN-8 (1 μmol/L) or SR3576 (25 µmol/L), lower levels of Bcl-2 protein and higher levels of Bax protein caused by MPP^+^ were suppressed, eventually increasing the Bcl-2/Bax ratio (Figure 4B).

### 3.5. Hispidin Attenuated MPP^+^-Induced Caspase Mediated Apoptotic Pathway Activation

**Figure 5:**
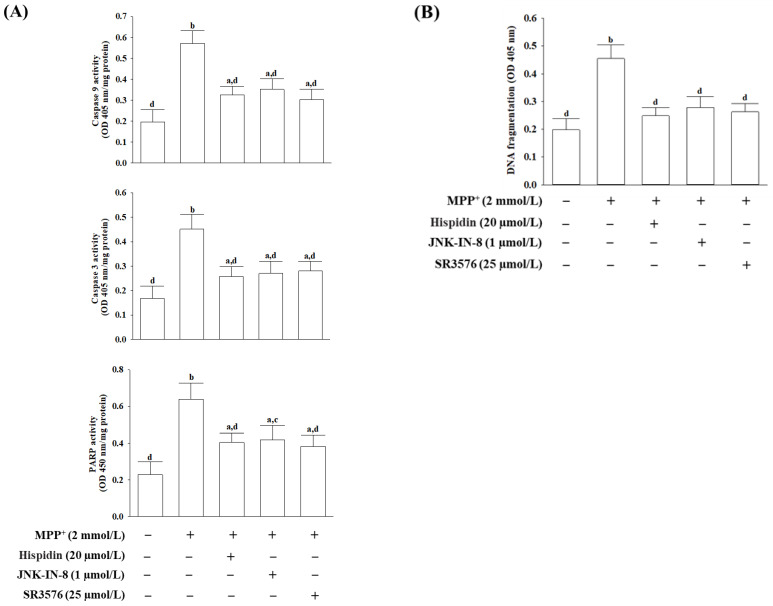
Hispidin reduces caspase-mediated apoptosis induced by MPP^+^. MS23.5 cells were pretreated with hispidin (20 µmol/L), JNK-IN-8 (1 µmol/L), or SR3576 (25 µmol/L) for 1 h and then exposed to 2 mmol/L MPP^+^ for another 24 h. (**A**) Caspase-9, caspase-3, and PARP activities were assessed using commercial colorimetric assay kits. (**B**) The extent of apoptosis was quantified using the ELISA kit to detect DNA fragments associated with cytoplasmic histones. The results are shown as the mean ± SD of five independent experiments (*n* = 5) performed in triplicate. ^a^ *p* < 0.05 and ^b^ *p* < 0.01 compared to the untreated control group. ^c^ *p* < 0.05 and ^d^ *p* < 0.01 compared to the untreated MPP^+^ group.

Casepase-9 and -3 activities detected in cells receiving MPP^+^-induction were respectively 2.9- and 2.6-fold greater than those of non-MPP^+^ stimulating cells (Figure 5A). Casepase-9 and -3 activities in the pretreated hispidin (20 μmol/L) group were lower to 57.1% and 56.4% of values for MPP^+^ induction, respectively (Figure 5A). When cells were pretreated with JNK-IN-8 (1 μmol/L) or SR3576 (25 µmol/L) following MPP^+^ induction, reduced caspase-9 activity by 38.4% and 40.1%, respectively. (Figure 5A). The high activity of caspase-3 in cells stimulated by MPP^+^ was reduced to 59.1% and 60.4%, respectively, in cells pretreated with JNK-IN-8 (1 μmol/L) or SR3576 (25 μmol/L; Figure 5A). PARP activity in MES23.5 cells exposed to MPP^+^ was 2.8-fold higher than that of the control. Either hispidin (20 μmol/L) or JNK-IN-8 (1 μmol/L) and SR3576 (25 μmol/L) decreased PARP activity in MPP^+^ exposed cells by 35.7, 34.6 and 39.2%, respectively (Figure 5A).

The fragmentation of apoptotic DNA in MPP^+^-induced MES23.5 cells increased to 2.3-fold of the control, which was decreased by 43.2, 38.8, and 42.2% under cells receiving pretreatment with hispidin (20 μmol/L), JNK-IN-8 (1 μmol/L), and SR3576 (25 μmol/L), respectively (Figure 5B).

## 4. Discussion

The precise etiology of the degeneration and death of dopaminergic neurons in PD remains uncertain; the exacerbation of oxidative stress is considered a major contributor to the damage of dopaminergic neurons in the pathogenesis of PD [1]. When the toxic metabolite of MPTP, MPP^+^, continues to accumulate in the synaptosomal vesicles of dopaminergic neurons, it causes mitochondrial dysfunction, oxidative stress, and programmed cell death that simulates parkinsonian syndrome in cellular and animal models [20]. Cell viability was first assessed to determine if hispidin saves cells from MPP^+^-induced neurotoxicity was associated with combating oxidative stress. Our results demonstrated that hispidin acts as a cytoprotective on MPP^+^-exposed MES23.5 cells as a result of reduced ROS production and improved cell viability. This is consistent with previous evidence that hispidin was protective for cells exposed to hydrogen-peroxide-induced oxidative stress [26].

Even though ROS can cause oxidative damage to biomolecules at tightly regulated levels, they are needed to sustain redox cell homeostasis and are involved in adaptive signaling to overcome various stresses in a way that supports health [27]. If the antioxidant system is unable to maintain ROS under control, high levels of ROS may cause oxidative stress leading to the activation of malignant signaling or cell death [27]. Although antioxidant defense, including SOD, CAT, and GSH-Px, can scavenge ROS and reduce the oxidation of cellular molecules, thus against the deleterious effects of various oxidative stress, these antioxidants appear insufficient if higher levels of ROS activate cell death processes [4]. MPP^+^-induced ROS accumulation in MES23.5 cells was accompanied by a clear decline in SOD, CAT, and GSH-Px activity, providing that MPP^+^-inducing oxidative stress is associated with a disruption of the initial antioxidant defense systems [20]. The present study indicated that pretreatment with hispidin abolished the MPP^+^-induced inhibition of SOD, CAT, and GSH-Px activities in MES23.5 cells. These results suggest that the protective effect of hispidin against MPP^+^-induced neurotoxicity may be mediated by the enhancement of the first-line antioxidant defense of scavenging different radicals, thereby attenuating oxidative damage. Because DA is easily oxidized, the oxidative destruction in dopaminergic neurons leading to lower cellular DA content is central to PD development; antioxidants can, of course, protect neurons against oxidative damage to destroy dopaminergic neurons in PD [28]. In this study, DA levels decreased after MPP^+^ stimulation, but preconditioning hispidin significantly increased DA levels in MPP^+^-stimulated MES23.5 cells. This could be due to the antioxidant effect of hispidin, which protects DA against oxidation as well as maintains cellular function.

MPP^+^ exerts its neurotoxicity mainly by inhibiting the activity of mitochondrial respiratory chain complexes I [29]. In the current study, we observed that hispidin could restore mitochondrial respiratory chain complexes I activity, suggesting that the restoration of mitochondrial respiratory chain complexes I activity might contribute to the neuroprotective effects of hispidin. In order to verify this hypothesis, further investigations on hispidin blocking the concentration of MPP^+^ into mitochondria or competitively combined to hydrophobic binding site on NADH dehydrogenase need to be further clarified.

Once MPP^+^ damages mitochondrial respiratory enzymes, mitochondrial respiration becomes dysfunctional, and the potential of the mitochondrial membrane decreases, leading to a reduction in ATP production and increasing mitochondrial permeability [29]. It is well known that the Bcl-2 family of proteins plays an important role in intracellular apoptotic signal transduction by regulating the permeability of the mitochondrial membrane [30]. Among members of the Bcl-2 family, Bax can especially regulate the permeability of the outer mitochondrial membrane, leading to increased cytochrome c release from mitochondria to trigger apoptosis cascades [30]. Bcl-2 acts as an inhibitor of mitochondrial permeability by changing the conformation within the mitochondrial membrane to bind the Bax and block the oligomerization of the Bax [31]. The Bcl-2/Bax ratio is regarded as a better predictor of apoptosis than either Bcl-2 or Bax alone [32]. Cytochrome c in the cytosol participates in caspase activation via binding to Apaf-1, and caspase 9 forms the apoptosome that activates downstream effector caspases 3 to conduct the process of apoptosis [33]. Activated caspase-3 can also cleave PARP-1, which could suppress DNA repair and facilitate caspase-mediated DNA fragmentation; this phenomenon has been proven in several neurological diseases [34]. Mitochondrial therapies targeted at restoring mitochondrial function and promoting the survival of neuronal cells are tendencies of drug development in the prevention or treatment of neurodegeneration [35]. The mitochondrial-mediated apoptotic phenomenon has been shown in MES23.5 cells exposed to MPP^+^, as shown by mitochondrial membrane potential loss, ATP depletion, cytochrome c release, cleavage, and activation of initiator caspase-9, effector caspase-3, and PARP proteolysis. Down-regulation of the Bcl-2/Bax ratio, coupled with significant DNA fragmentation, was also evidence of apoptosis in MPP^+^-exposed MES23.5 cells. All of the above events associated with MPP^+^-induced mitochondria-dependent caspase cascades were attenuated under cells receiving hispidin pretreatment. Hispidin could therefore be seen as being able to target mitochondrial defects from oxidative damage to prevent neuronal death and enhance existing cellular function leading to reducing the risk of neurodegeneration.

Neurotoxins and oxidative stress have been shown to phosphorylate JNK and soon afterward activate multiple apoptosis-related transcriptional factors such as c-Jun [36]. JNK-mediated apoptosis also through effects stimulates the expression of pro-apoptotic genes and reduces the expression of pro-survival genes [37]. In addition to regulating the transcription of genes associated with apoptosis, JNK also, through transcription-independent mechanisms, mediates apoptosis [37]. Inhibition of abnormal c-JNK signaling overactivation can therefore prevent cell death, making JNK a promising target for extending pharmacological intervention [37]. Comparisons with JNK1 and JNK2 are expressed throughout the body; JNK 3 is mainly expressed within the nervous system [38]. Therefore, JNK3 inhibitors have been identified as a potential therapeutic target for neurodegenerative diseases, although they are not yet in clinical use [38]. Hispidin has the same tendency as a pan-JNK inhibitor JNK-IN-8 or JNK3 inhibitor SR3576 to mitigate mitochondrial dysfunctions and subsequent activation of the intrinsic apoptotic pathway in MPP^+^-exposed MES23.5 cells, while SR3576 seems to be more efficient. In fact, hispidin could not completely block the MPP^+^-induced mitochondrial ROS and the intrinsic apoptotic pathway effectors. Hispidin contributes to the partial protection against neurons from MPP^+^-induced impairments by improving the mitochondrial JNK pathway can be considerable. In addition, considering that the most prominent mitochondrial inducers of JNK signaling ROS, the neuroprotective effect of hispidin is that it is an antioxidant capable of removing ROS, which indirectly results in the inactivation of the JNK pathway, or being a primary blockage target at JNK requires further study to clarify.

ELISA showing excellent quantitative features with reproducibility is often used to quantify a specific protein that exists in a mixture of different proteins [39]. The ELISA is therefore considered more valid for accurate quantification of quantitative change. Our study aimed to investigate the quantitative changes of endogenous proteins depending on the revealed activation degrees in MPP^+^-mediated ROS-dependent apoptosis, including the JNK-Bax-caspase-3 pathway in MES23.5 cells by hispidin pretreatment. Using ELISA enables qualitative analysis to be considered an appropriate technique for the experiment depending on the objective of the study.

Although our study proved the neuroprotective role of hispidin in MPP^+^-induced MES23.5 cells, an in vitro PD-like model, the feasibility of clinical application requires further evaluation. Generally, molecules can cross the blood–brain barrier (BBB) and could be used for brain disorders [40]. Research on CNS disease-modifying treatments has given rise to a cemetery of ineffective drugs, discarded in part because of their inability to cross the BBB [40]. Developing effective delivery strategies to address the issue of transporting drugs to BBB remains a challenge in the treatment of neurological diseases [41]. In general, only lipophilic molecules of low molecular weight (less than 400 Da) and positive charge may cross the BBB [42]. Based on structural analysis, phenolic compounds containing at least one hydroxy group that is directly bound to an aromatic ring, thus theoretically, be not easy to cross the BBB [43]. Hispidin actually contains more than one hydroxyl group and is not small enough to cross the BBB [44]. Modifying hispidin to improve lipid solubility through the addition of lipid groups or functional groups to the polar ends of molecules can be useful in improving across BBB [41]. Additionally, hispidin can be positively charged with a cationic molecule for further penetration into BBB for in vivo use [41].

Since PD is a chronic disease that requires long-term therapy, further assessment of the safety of hispidin becomes a critical issue. In a toxicity study, it demonstrated that mushroom mycelium enriched with 3 mg/g of hispidin has very low toxicity based on the results obtained in the Ames test, in vitro chromosome aberration test, acute oral toxicity test, and bone marrow micronucleus test [45]. Hispidin could be regarded as a bioactive compound used safely for a therapeutic strategy on neural disorders and other diseases [9].

The effect of pretreatment of hispidin against MPP^+^-induced cell lesions in MES23.5 cells was clarified in this study. Although this discovery provides new insights into the prophylactic impact of hispidin on neural degeneration, it is unclear whether hispidin can play a therapeutic role after MPP^+^-induced neuronal damage, giving a new direction to our future study.

In conclusion, our results conferred a neuroprotective role on hispidin against the MPP^+^-induced cell model. This neuroprotective impact of hispidin may be attributed to the restoration of the mitochondrial complex respiratory chain I activity to improve mitochondrial dysfunction in addition to its potent antioxidant capacity. The downregulation of oxidation-dependent JNK signaling thus leads to caspase cascade suppression, which is the mediated mechanism of hispidin against MPP^+^-triggered apoptosis in MES23.5 cells. Our findings suggest that hispidin may represent a new therapeutic strategy for preventative and/or complementary PD therapies.

## Data Availability

All the data needed to evaluate the conclusions in the paper are present in the paper. Additional data related to this paper may be requested from the authors.
